# Optimizing positive end-expiratory pressure by oscillatory mechanics minimizes tidal recruitment and distension: an experimental study in a lavage model of lung injury

**DOI:** 10.1186/cc11858

**Published:** 2012-11-07

**Authors:** Emanuela Zannin, Raffaele L Dellaca, Peter Kostic, Pasquale P Pompilio, Anders Larsson, Antonio Pedotti, Goran Hedenstierna, Peter Frykholm

**Affiliations:** 1 TBM Lab, Dipartimento di Bioingegneria, Politecnico di Milano, Piazza Leonardo da Vinci 32, 20133 Milano, Italy; 2 Department of Surgical Sciences, Anaesthesia and Intensive Care, Uppsala University, S 751 85 Uppsala, Sweden; 3 Department of Medical Sciences, Clinical Physiology, Uppsala University, 751 85 Uppsala, Sweden

## Abstract

**Introduction:**

It is well established that during mechanical ventilation of patients with acute respiratory distress syndrome cyclic recruitment/derecruitment and overdistension are potentially injurious for lung tissues. We evaluated whether the forced oscillation technique (FOT) could be used to guide the ventilator settings in order to minimize cyclic lung recruitment/derecruitment and cyclic mechanical stress in an experimental model of acute lung injury.

**Methods:**

We studied six pigs in which lung injury was induced by bronchoalveolar lavage. The animals were ventilated with a tidal volume of 6 ml/kg. Forced oscillations at 5 Hz were superimposed on the ventilation waveform. Pressure and flow were measured at the tip and at the inlet of the endotracheal tube respectively. Respiratory system reactance (Xrs) was computed from the pressure and flow signals and expressed in terms of oscillatory elastance (E_X5_). Positive end-expiratory pressure (PEEP) was increased from 0 to 24 cm H_2_O in steps of 4 cm H_2_O and subsequently decreased from 24 to 0 in steps of 2 cm H_2_O. At each PEEP step CT scans and E_X5 _were assessed at end-expiration and end-inspiration.

**Results:**

During deflation the relationship between both end-expiratory and end-inspiratory E_X5 _and PEEP was a U-shaped curve with minimum values at PEEP = 13.4 ± 1.0 cm H_2_O (mean ± SD) and 13.0 ± 1.0 cm H_2_O respectively. E_X5 _was always higher at end-inspiration than at end-expiration, the difference between the average curves being minimal at 12 cm H_2_O. At this PEEP level, CT did not show any substantial sign of intra-tidal recruitment/derecruitment or expiratory lung collapse.

**Conclusions:**

Using FOT it was possible to measure E_X5 _both at end-expiration and at end-inspiration. The optimal PEEP strategy based on end-expiratory E_X5 _minimized intra-tidal recruitment/derecruitment as assessed by CT, and the concurrent attenuation of intra-tidal variations of E_X5 _suggests that it may also minimize tidal mechanical stress.

## Introduction

Current strategies for mechanical ventilation of patients with acute respiratory distress syndrome (ARDS) include low tidal volumes and increased positive end-expiratory pressure (PEEP) [1]. The physiological basis for these strategies is still under debate, but may involve the reduction of forces of stress and strain on the parenchyma [2]. The ARDSNet protocol is a table-based approach of setting PEEP and the fraction of inspired oxygen (FIO_2_) levels to achieve an oxygenation target [3]. The achieved PEEP levels may, however, generate tidal recruitment/derecruitment, which has been identified as an important source of mechanical stress and damage to the lung parenchyma [4-6], as well as tidal and expiratory overdistension in the non-dependent regions of the lung in patients with low potential for recruitment [7].

Several strategies for optimizing PEEP based on lung mechanics have been evaluated in animal models with promising results [8-11]. However, all of them are based on the minimization of the mean value of either lung elastance (or similarly on the maximization of the mean value of compliance) or the degree of heterogeneity of lung mechanical properties computed over the whole breath without considering intra-tidal phenomena. In order to avoid tidal recruitment/derecruitment, PEEP should be set at the minimum level that counteracts derecruitment of the lung at end-expiration. Thus, at steady state, if no derecruitment occurs during expiration, no intra-tidal recruitment will occur during inspiration. In order to achieve this, PEEP must be higher than that titrated by standard techniques based on the assessment of respiratory mechanics over the whole breath. On the other hand, high PEEP levels together with large, or even moderate, tidal volumes may cause end-expiratory overinflation, promoting tidal overdistension during each breath and cyclic mechanical stress to the lung parenchyma.

The forced oscillation technique (FOT) is a non-invasive technique that allows the measurement of pulmonary mechanical properties at a given lung volume, independent of tidal volume and spontaneous breathing, with high temporal resolution. Briefly, it consists of evaluating the response of the respiratory system to small amplitude pressure oscillations in terms of impedance (Zrs). FOT has been successfully applied in humans during both invasive [12-16] and non-invasive mechanical ventilation [17-19]. Zrs is made up of two terms, resistance (Rrs) and reactance (Xrs). The latter is related to the dynamic elastance (the inverse of compliance, reflecting tissue elasticity, size of the lung, amount of alveolar units connected to the airway opening) and inertia (reflecting the energy that has to be spent to accelerate gas and tissues) of the respiratory system.

Zrs also depends on the frequency at which it is assessed and its frequency dependence is more marked in presence of heterogeneities. However, we have recently shown that Xrs measured at the oscillatory frequency of 5 Hz is strongly related to the fraction of recruited tissue irrespective of its spatial distribution [20] and is effective in guiding PEEP titration through the identification of the optimal trade-off between recruitment and lung tissue distention in experimental acute lung injury [21,22].

This study is an extension of a previous study [21] that combined end-expiratory computed tomography (CT) and FOT data. The present study also includes end-inspiratory FOT and CT data to enable the analysis of intra-tidal changes in lung mechanics and aeration in order to determine the best PEEP setting that would minimize cyclic recruitment and distention of lung parenchyma.

## Materials and methods

Six healthy pigs (weight 24.5 to 29 kg, Swedish mixed country breed) were studied at the Department of Surgical Sciences, Hedenstierna Laboratory and the Department of Radiology of the University Hospital of Uppsala, Sweden. The study was approved by the Uppsala University Animal Ethics Committee.

### Animal preparation

Anesthesia was induced by tiletamine 6 mg·kg^-1^, zolazepam, 6 mg·kg^-1^, xylazine 2.2 mg·kg^-1 ^i.m., maintained with an infusion of phenobarbital 1 mg/ml, pancuronium 0.032 mg/ml and morphine 0.06 mg·ml^-1 ^at a rate of 8 ml·kg^-1^·h^-1^. After a bolus injection of fentanyl 10 μl·kg^-1 ^the animal was tracheotomized and ventilated through a shortened 8 mm endotracheal tube (ETT) (Mallinckrodt, Athlone, Ireland). The animal was ventilated in volume control mode (Servo *i *ventilator, Maquet, Solna, Sweden) with a tidal volume of 6 ml/kg of body weight, a pretrial PEEP of 6 cm H_2_O, and the respiratory rate titrated to obtain normocapnea. FIO_2 _was kept at 1.0 for the duration of the experiment.

Lung injury was induced by repeated bronchoalveolar lavage with warm saline. The end-point of the lavage was a sustained reduction in the partial pressure of oxygen in arterial blood (PaO_2_)/FIO_2 _<100 mmHg during a period of 60 minutes.

### Measurements

Systemic and pulmonary arterial pressures, heart rate, mixed venous saturation, and body temperature were continuously monitored (CCOmbo 7.5-Fr, Edwards Life Sciences LLC, Irvine, CA, USA). FOT was applied with a system described elsewhere [20]. Briefly, low amplitude sinusoidal pressure oscillations (approximately 1.5 cm H_2_O peak-to-peak) at 5 Hz were generated by a loudspeaker connected to the inspiratory line of the ventilator. Flow at the airway opening ($\dot{\text{V}}\text{ao}$) was measured by a differential pressure transducer (PXLA02X5DN, Sensym, Milpitas, CA, USA) connected to a mesh-type heated pneumotachograph. Pressure (Ptr) was measured at the tip of the endotracheal tube by a differential pressure transducer (PXLA0075DN, Sensym, Milpitas, CA, USA). All signals were sampled at 200 Hz.

### Experimental protocol

After preparation, the animal was positioned in a CT scanner and connected to the FOT ventilator system. PEEP was increased from 0 to 24 cm H_2_O in steps of 4 cm H_2_O and subsequently decreased from 24 to 0 in steps of 2 cm H_2_O. Since optimal PEEP is defined during a decremental PEEP trial after lung recruitment [23] the incremental series was performed in large steps because the main aim was to achieve full recruitment, while during the deflation series PEEP was reduced by smaller steps in order to define the optimal value with higher resolution. The duration of each step was eight minutes.

During each step, after four minutes a CT scan was performed during an inspiratory hold and, after a few breaths, it was repeated during an expiratory hold (by using the inspiratory and expiratory hold functions available in the ventilator, which resulted in no flow and pressure constant to Plateau pressure and PEEP respectively). The total duration of the experiment was approximately 150 minutes. End-expiratory CT and FOT data has been published in a previous paper [21].

### Data analysis

*Lung mechanics: *respiratory system input reactance (Xrs) was calculated from $\dot{\text{V}}\text{ao}$ and Ptr by a least squares algorithm [24,25] and used to compute oscillatory elastance (the inverse of oscillatory compliance defined in [21], E_X5 _= 1/C_X5_) with the following equation:

(1) $$E_{\text{x}5}=-2\times \pi \times 5\times \text{Xrs}$$

E_X5 _changes were computed for the full duration of the experiment. For optimal comparisons of FOT and CT data, inspiratory and expiratory E_X5 _values were averaged from the initial parts of the inspiratory and expiratory holds respectively.

Dynamic elastance and resistance (Rdyn) were calculated by fitting Ptr and $\dot{\text{V}}\text{ao}$ to the equation of motion of the respiratory system:

$$\text{Ptr}=\text{Edyn}\times \text{V}+\text{Rdyn}\times \dot{\text{V}}\text{ao}+\text{EEP}$$

where V is volume obtained by integration of $\dot{\text{V}}\text{ao}$ and EEP is the end-expiratory pressure. The fitting was performed by the least squares method on approximately five to ten breaths preceding the CT scans.

Intra-tidal changes in Edyn were evaluated using the SLICE method [26,27]. The range between 10 and 90% of the inspiratory signals was analyzed at six subsequent volume steps. Elastance was computed as described above for the lowest (E_LOW_) and the highest (E_HIGH_) volume steps and compared to end-expiratory and end-inspiratory E_X5 _respectively.

*Computed tomography analysis: *Changes in lung aeration were studied by analyzing whole-body CT scans (Somatom Sensation 16, Siemens, Forchheim, Germany). CT rotation time was 0.5 sec at effective 100 mA, 120 kV, collimation 16 × 0.75 and pitch 1.05. The CT exposure started immediately at end-expiration or end-inspiration and moved from apex to base. A full spiral CT took approximately 10 sec. Images were reconstructed with 8 mm slice thickness using a standard reconstruction filter (B41f, Siemens). The images were analyzed using dedicated software (Maluna, version 2.02, Mannheim, Germany). The lung contours were manually traced in all slices to define the regions of interest. The total lung volume was subdivided into over-aerated (OA, -1,000 to -900 Hounsfield units, HU), normally aerated (-900 to -500 HU), poorly aerated (PA, -500 to -100 HU) and non-aerated (NA, -100 to +100 HU) volumes as suggested previously [28,29]. Lung gas (Vgas) and tissue (Vtiss) volumes were calculated using standard equations [28] for both the whole lung and for each aeration compartment.

The volume of tissue in the non-aerated region expressed as a percentage of total lung tissue (VtissNA%), which is equivalent to the weight of the non-aerated region expressed as a percentage of total lung weight, was used as an index of derecruitment. The difference between end-expiratory and end-inspiratory VtissNA% was used to quantify intra-tidal recruitment/derecruitment.

### Statistical analysis

Data is expressed as mean ± SD. Significance of differences between end-inspiration and end-expiration was tested by two-way ANOVA for repeated measurements. Multiple comparisons after ANOVA were performed using the Holm-Sidak test. Differences were considered statistically significant for *P *<0.05.

## Results

**Figure 1:**
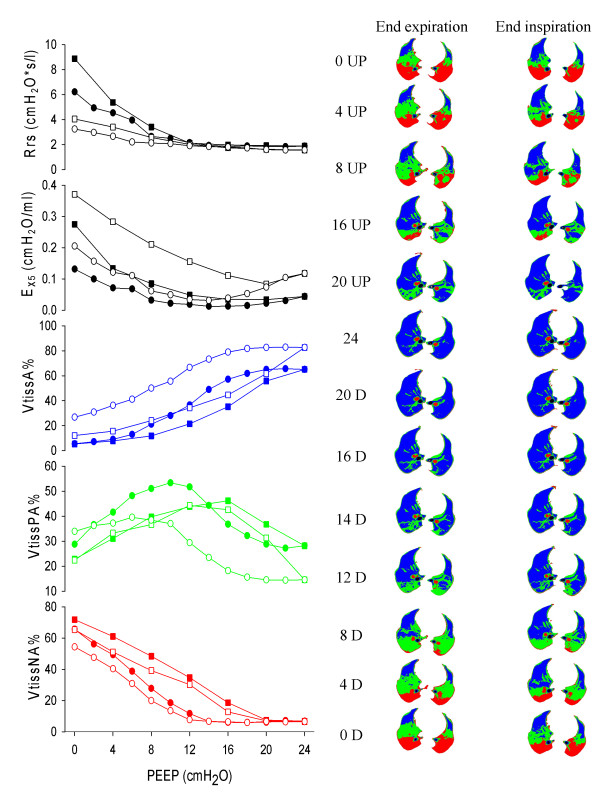
**Left panel: Respiratory system resistance (Rrs), oscillatory elastance (E_X5_, the inverse of compliance), percentage volume of aerated (VtissA%, blue), poorly aerated (VtissPA%, green) and nonaerated (VtissNA%, red) tissue at end-expiration (closed symbols) and at end-inspiration (open symbols) for one representative animal**. Squared symbols are used for incremental positive end-expiratory pressure (PEEP), circles for decremental. Right panel: a representative computed tomography (CT) slice (selected approximately 1 cm above the diaphragmatic dome) of the same animal at end-expiration (EE) and at end-inspiration (EI), at the different PEEP levels during the incremental (UP) and decremental (D) PEEP trial. Colour code for regions as above.

Figure 1 shows resistance (Rrs), oscillatory elastance (E_X5_) and the volumes of differently aerated compartments assessed by CT for a representative pig during the incremental/decremental PEEP trial.

**Figure 2:**
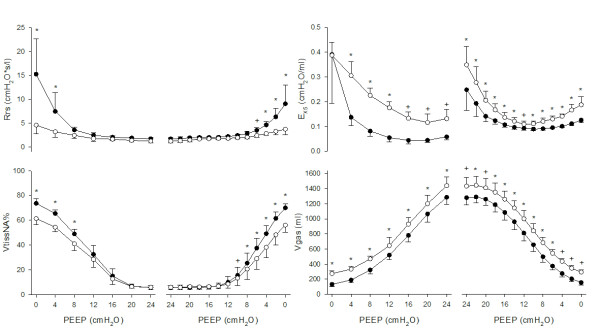
**Respiratory system resistance (Rrs), oscillatory elastance (E_X5_), percentage volume of non-aerated tissue (VtissNA%), lung gas volume (Vgas) at end-expiration (closed symbols) and at end-inspiration (open symbols) during the incremental and decremental positive end-expiratory pressure (PEEP) trial as a function of PEEP**. Data are reported as mean and SD. Significance of differences between inspiration and expiration: *P <0.01, +*P *<0.05.

Figure 2 shows mean data from all pigs. As expected, Rrs decreased with increasing PEEP. The relationship between E_X5 _and PEEP was U-shaped both at end-expiration and at end-inspiration. E_X5 _was higher (higher tension applied to the tissues, lower compliance) at end-inspiration (0.15 ± 0.10 cm H_2_O/ml) than at end-expiration (0.08 ± 0.90 cm H_2_O/ml) and during incremental PEEP steps than during the decremental PEEP series. Both end-inspiratory and end-expiratory E_X5 _reached their minimum values (maximum values of compliance) during the deflation series at a PEEP level of 12 and 14 cm H_2_O respectively. The difference between end-inspiratory and end-expiratory E_X5 _was higher during the incremental than during the decremental PEEP series and reached its minimum during deflation at a PEEP level of 12 cm H_2_O.

As expected, the percentage volume of aerated tissue (VtissA%) had a sigmoidal shape against PEEP and presented hysteresis. The PEEP level corresponding to the minimum end-expiratory E_X5 _(maximal compliance), defined as the open lung PEEP (PEEPol) [21], was 13.4 ± 1.0 cm H_2_O (mean ± SD). The minimum of end-expiratory E_X5 _occurred at a lower PEEP value, 13.0 ± 1.0 cm H_2_O. E_X5 _was significantly higher at end-inspiration than at end-expiration, indicating higher tissue distension. The difference between end-expiratory and end-inspiratory E_X5 _(related to the cyclic distension applied to the aerated tissues) was lower during the decremental PEEP steps and reached a minimum at the PEEP value of 12 cm H_2_O.

**Figure 3:**
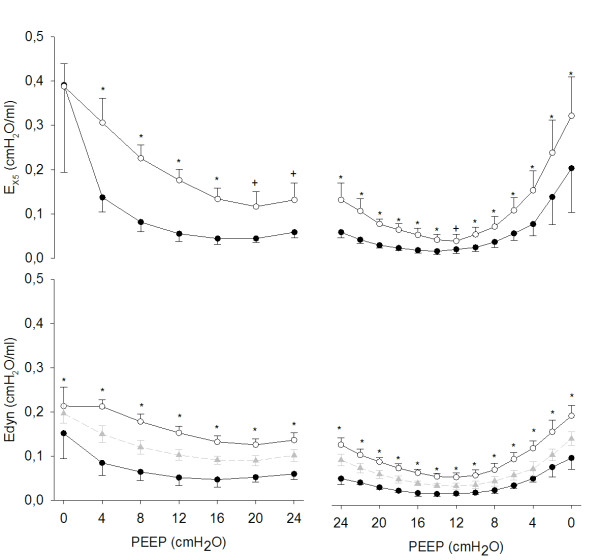
**End-inspiratory (open symbols) and end-expiratory (closed symbols) oscillatory elastance (E_X5_) and dynamic elastance (Edyn)**. Edyn has been computed over the whole breath (grey triangles, dashed line) and intra-tidal, at the highest (open circles, solid lines) and at the lowest (closed circles, solid line) portion of tidal volume. Data are reported as mean and SD. Significance of differences between inspiration and expiration: *P <0.01, +*P *<0.05.

Figure 3 shows tidal elastance (Edyn) computed over the whole breath as well as at the lowest (E_LOW_) and highest (E_HIGH_) segments of the tidal volume (see Methods) for comparison with end-expiratory and end-inspiratory E_X5 _respectively. Like E_X5_, both E_HIGH _and E_LOW _displayed U-shaped relationships with PEEP, with the minimum occurring at 12 cm H_2_O for E_HIGH _and at 14 cm H_2_O for E_LOW_. E_HIGH _was always lower than E_LOW _and the difference was minimal at 12 cm H_2_O similarly to intra-tidal E_X5. _Edyn computed over the whole breath stayed in between E_LOW _and E_HIGH _and was minimal at 12 cm H_2_O.

Even though Edyn and E_X5 _displayed a similar relationship vs. PEEP and similar changes within the respiratory cycle, the absolute values of E_X5 _were on average higher than those of Edyn. This difference is related to the different frequencies at which elastance was assessed: the respiratory frequency (approximately 0.5 Hz) for Edyn and 5 Hz for E_X5_. Moreover Rdyn, assessed at the breathing frequency, was always higher that Rrs assessed by FOT at 5 Hz. The relationship between Edyn-E_X5 _and PEEP was dome-shaped, the relationship between Rdyn-Rrs and PEEP was U-shaped and the absolute values of these differences reached a minimum during deflation at PEEP = 8 and 10 cm H_2_O respectively.

**Figure 4:**
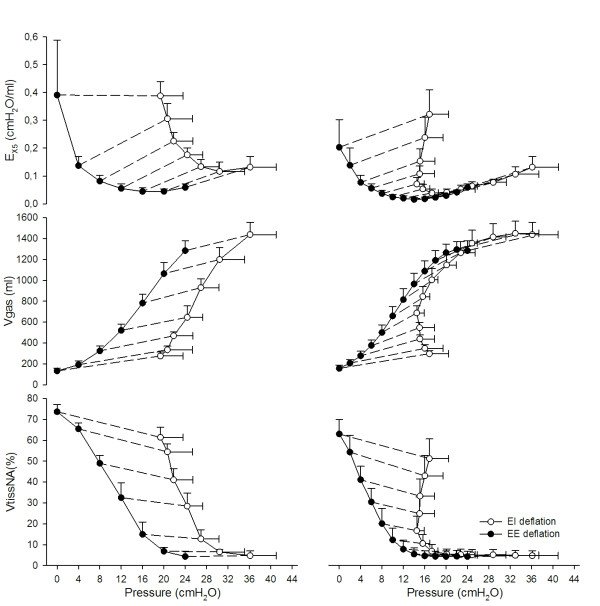
**Oscillatory elastance (E_X5_), gas volume (Vgas) and percentage volume of non-aerated tissue (VtissNA%) as a function of pressure during the incremental (left panel) and decremental (right panel) positive end-expiratory pressure (PEEP) trial**. Open symbols refer to end-inspiratory data points, closed symbols to end-expiratory ones. Dashed lines connect end-inspiration and end-expiration at the same PEEP step.

Figure 4 shows E_X5_, Vgas and VtissNA% as a function of pressure, displaying differences in lung mechanics and aeration between end-inspiration and end-expiration at comparable pressure levels.

## Discussion

FOT has all the characteristics needed to provide a bedside tool that allows the assessment of lung mechanics with high temporal resolution and at a given lung volume (not averaging over a breath) without being affected by the non-linearities of the respiratory system; therefore, it can be used to measure lung mechanical properties at both end-inspiration and end-expiration, possibly helping to separate the effects of PEEP and tidal volume. The present study uses data already acquired from a previous study [21] with the addition of end-inspiratory FOT and CT data. The analysis of intra-breath changes of lung mechanics and aeration at different PEEP levels led to the following novel finding in addition to what has already been reported: in a lavage model of surfactant-depletion the PEEP level that minimized end-expiratory E_X5 _(or similarly the PEEP level that maximized end-expiratory compliance, C_X5_) 1) was the lowest PEEP that avoided intra-tidal recruitment/derecruitment as assessed by CT; 2) minimized intra-tidal changes in E_X5_, suggesting that tidal tissue distension applied to the lung was also minimized.

In theory, also Cdyn and Edyn can be computed at various points of the tidal volume using the SLICE method [26,27,30], which has recently been improved by the gliding [31] and the adaptive [32] slice methods that allow a more detailed and less noisy description of intra-tidal lung compliance during fully controlled ventilation. Figure 3 shows that, when the subject is paralyzed as in the present study, the intra-breath analysis of E_X5 _and of Edyn provides very similar information. However, Edyn (and similarly Cdyn) is strongly affected by spontaneous breathing and the SLICE method is in theory even more susceptible to these artifacts because the fitting is performed on a small volume variation.

We cannot from this study assess the usefulness of FOT during spontaneous breathing since the animals were paralyzed in order to perform CT scans at fixed lung volumes. However, several studies have shown reliable results of lung mechanics by FOT measurements also in the presence of spontaneous activities of the patients, such as in quiet breathing [33-35], CPAP [17] and non-invasive mechanical ventilation in COPD patients [36,37].

### Impedance data interpretation

We have previously shown that Xrs reflects both lung volume recruitment and tissue distension so that its maximum end-expiratory value during a decremental PEEP trial identifies the lowest PEEP needed to keep the lung open at end-expiration (PEEPol) [21]. Moreover, the use of end-expiratory Xrs to set PEEP led to a more protective ventilation strategy compared with the ARDSNet approach [22]. PEEPol can be defined as the PEEP level that minimizes end-expiratory E_X5. _We now found that PEEPol is the lowest level of PEEP that inhibits intra-tidal recruitment/derecruitment. In addition, we evaluated E_X5 _at end-inspiration and found that it was always higher than at end-expiration. Since recruitment is associated with a decrease in E_X5 _while parenchymal distension leads to an increase in E_X5_, the higher values of E_X5 _found during inspiration compared with expiration suggest that in this model of lung injury the impact of intra-tidal distension on intra-tidal changes in E_X5 _dominates over that of intra-tidal recruitment/derecruitment.

The relationship between end-inspiratory E_X5 _and PEEP could be described by a U-shaped curve, which was similar to the one between end-expiratory E_X5 _and PEEP. The difference between the two (which can be considered an indicator of tidal stress) was higher both at very low and at very high PEEP levels compared with the optimal PEEP (Figure 2 and 3). At the beginning of the decremental PEEP trial both end-inspiratory and end-expiratory pressures are able to keep the lung recruited. As PEEP approaches closing pressure, end-expiratory E_X5 _reaches its minimum (in this study this occurred, on average, at 14 cm H_2_O). In the PEEP range between the minima of end-inspiratory and end-expiratory E_X5 _the lung is partially derecruited at end-expiration but it is still fully recruited at end-inspiration. This means that cyclic recruitment/derecruitment starts to develop, as confirmed by CT data. For PEEP values lower than the one that minimizes end-inspiratory E_X5_, the lung is partially collapsed even at end-inspiration. This is associated with increasing difference between end-expiratory and end-inspiratory E_X5 _(which may indicate increasing tidal stress) and increasing cyclic recruitment/derecruitment (confirmed by CT). Therefore, the fact that intra-tidal changes in E_X5 _are minimized around PEEPol suggests that ventilating either a partially collapsed or an over-aerated lung is associated with increased cyclic stress.

This interpretation is supported by the relationship between E_X5 _and pressure (Figure 4). During the decremental PEEP trial, for PEEP levels equal to or greater than 14 cm H_2_O both the end-inspiratory and the end-expiratory E_X5 _points lay on the same line. This suggests that in that range of pressures E_X5 _is mostly affected by the elastic characteristics of the lung and not by recruitment/derecruitment of alveolar units, as confirmed by VtissNA% assessed by CT. As PEEP is further reduced, the inspiratory and expiratory curves diverge, indicating that the lung starts to derecruit.

Our data also shows that changes in E_X5 _reflect changes in Edyn. However their absolute values are slightly different, which is expected because they are assessed at different frequencies: the respiratory frequency (approximately 0.5 Hz) for Edyn and 5 Hz for E_X5_. Moreover Rdyn, assessed during tidal breathing, was always higher than Rrs assessed at 5 Hz. The frequency dependence of resistance and elastance is a consequence of heterogeneity of time constants [38-40]. The frequency dependence that we observed is in agreement with that reported in previous studies in which PEEP titration was guided by the minimization of heterogeneities [8,41]. In the present study, the absolute values of Edyn-E_X5 _and Rdyn-Rrs were exaggerated during the inflation series and at low PEEP levels, in the presence of more heterogeneous collapse of lung tissue [8,41]. Therefore, the PEEP optimization strategy based on the trade-off between derecruitment and overdistension was in agreement with that based on the reduction of heterogeneities.

Finally, this work gives further evidence to the significance of titrating PEEP during a decremental PEEP trial. In fact during the decremental series intra-tidal recruitment/derecruitment (Figure 4) and distension (Figure 3 and 4) are reduced compared with the incremental one.

### Limitations of the study

In the present study the internationally recommended thresholds were used for separating differently aerated regions [28,42-45]. We used a cut-off density between the aerated and the over-aerated region of -900 HU similar to Vieira *et al. *[29]. In contrast to other studies using similar animal models [10,46], we found a negligible over-aerated volume. To check whether this difference could be due to higher slice thickness, in a subset of pigs images were reconstructed both with 8 mm and with 1 mm thickness but this did not lead to differences in the message of our study. On the other hand, the mechanical distension detected by E_X5 _may not be directly related to over-aerated volume. In fact, over-aerated volume identified by CT represents lung regions overfilled with gas, while overdistension is defined as an excessive mechanical stress [28].

CT scans were performed during breath holds, which means that they represent the average picture of the lung over approximately 10 seconds, but it is possible that dynamic phenomena occurred during the measurements. On the contrary, E_X5 _data was extracted at the very beginning of the pauses and therefore they more closely represent what happens during tidal ventilation.

A limitation of this method, like others based on measurements performed at the airway opening, is that it only gives an average view of the mechanical properties of the lung. It is well established that the lung even in healthy conditions exhibits heterogeneities in regional ventilation [47], which increase in acute lung injury depending on the severity of the disease, PEEP and tidal volume [48]. These heterogeneities lead to regional differences in the stress-strain relationship [49], which cannot be taken into account by our method. On the contrary, FOT measurements performed by applying multi-frequency stimuli and fitting the data to proper mathematical models could account for mechanical heterogeneities of the injured lung [8,39,50].

Indeed, to facilitate the implementation of these results in clinical practice it will be necessary to integrate FOT into commercial mechanical ventilators and to infer the mechanical properties of the respiratory system from flow and pressure data measured prior to the ventilator tubing, which is a challenging issue that will be addressed in future works.

## Conclusions

In conclusion, since FOT allows the assessment of the mechanical properties of the respiratory system at any given lung volume, it can be used to evaluate elastance at end-inspiration and end-expiration. Optimizing PEEP by end-expiratory elastance minimizes intra-tidal recruitment/derecruitment with the potential to minimize cyclic mechanical stress on lung tissue. Moreover, the present data suggest that FOT measurements at end-inspiration could be useful also for optimizing tidal volume in order to further reduce harmful distension of the pulmonary structure, providing a tool for defining individualized protective ventilation settings. Future studies will be addressed at testing this hypothesis.

## Key messages

• FOT can be used to evaluate end-inspiratory and end-expiratory E_X5._

• In a lavage model of acute lung injury (ALI), the PEEP level that minimized end-expiratory E_X5 _was the lowest PEEP able to prevent intra-tidal recruitment assessed by CT.

• For PEEP levels at which E_X5 _was minimal intra-tidal changes in E_X5 _were also minimized, suggesting that they were associated with attenuated cyclic mechanical stress.

• Optimal PEEP based on E_X5 _also minimized intra-tidal changes in E_X5_, suggesting attenuated cyclic mechanical stress.

## Abbreviations

ALI: acute lung injury; ARDS: acute respiratory distress syndrome; CT: computed tomography; Edyn: dynamic elastance; ETT: endotracheal tube; E_HIGH_: dynamic elastance assessed at the highest portion of tidal volume; E_LOW_: dynamic elastance assessed at the lowest portion of tidal volume; E_X5_: oscillatory elastance; FIO_2_: fraction of inspired oxygen; FOT: forced oscillation technique; PaO_2: _partial pressure of oxygen in arterial blood; PEEP: positive end-expiratory pressure; PEEPol: open lung PEEP; Ptr: tracheal pressure; Rrs: respiratory system resistance; Vao: airway opening flow; Vgas: gas volume; Vtiss: tissue volume; VtissA%: percentage volume of aerated tissue; VtissNA%: percentage volume of non-aerated tissue; Xrs: respiratory system reactance; Zrs: respiratory system impedance.

## Competing interests

Politecnico di Milano University, the institution of EZ, RD, PP and AP, owns a patent on the use of forced oscillation technique for the detection of lung volume recruitment/derecruitment. The other authors have no competing interests to declare.

## Authors' contributions

EZ contributed to the study design, participated in the experimental activity, performed the data processing and contributed to the data interpretation and drafting of the manuscript. RD contributed to the study design, designed the experimental set-up, participated in the experimental activity and in the interpretation of the results and contributed to drafting the manuscript. PK contributed to the study design and participated in the experimental activity. PP designed the experimental set-up, participated in the experimental activity and contributed to data processing. AL contributed to the interpretation of results and critically revised the manuscript. AP contributed to the study design and to the interpretation of results. GH contributed to the study design and data interpretation and critically revised the manuscript. PF contributed to the study design, participated in the experimental activity and in the interpretation of the results and contributed to drafting the manuscript. All authors have read and approved the manuscript for publication.
